# Impact of very low carbohydrate ketogenic diets on cardiovascular risk factors among patients with type 2 diabetes; GRADE-assessed systematic review and meta-analysis of clinical trials

**DOI:** 10.1186/s12986-024-00824-w

**Published:** 2024-07-19

**Authors:** Parisa Ghasemi, Malihe Jafari, Saber Jafari Maskouni, Seyed Ahmad Hosseini, Roksaneh Amiri, Jalal Hejazi, Mahla Chambari, Ronia Tavasolian, Mehran Rahimlou

**Affiliations:** 1 Research Committee, Medical School, Arak University of Medical Sciences, Arak, Iran; 2 Department of Exercise physiology, Faculty of Physical Education and Sports Sciences, Allameh Tabataba’i University, Tehran, Iran; 3 Department of nutrition, School of Public health, Jiroft University of Medical Sciences, Jiroft, Iran; 4 Department of Nutrition, School of Allied Medical Sciences, Ahvaz Jundishapur University of Medical Sciences, Ahvaz, Iran; 5 Mofid Childrens Hospital Clinical Research Development Unit, Shahid Beheshti University of Medical Science, Tehran, Iran; 6 Department of Nutrition, School of Public Health, Zanjan University of Medical Sciences, Zanjan, Iran; 7 Department of Food Science and Nutrition, Faculty of Applied Sciences, UCSI University, 56000 Cheras, Wilayah Persekutuan Kuala Lumpur, Malaysia

**Keywords:** Type 2 diabetes, Very low carbohydrate ketogenic diets, T2DM, Meta-analysis

## Abstract

**Objective:**

This study was designed to evaluate the impact of VLCKD on cardiovascular risk factors in patients with T2DM.

**Methods:**

Until March 2024, extensive searches were conducted on PubMed, Scopus, Web of Science, Embase, and other relevant databases. The purpose was to identify clinical trials examining the impact of VLCKD on glycemic control, lipid profile, and blood pressure. The GRADE (Grading of Recommendations Assessment, Development, and Evaluation) method was used to assess the evidence’s degree of certainty.

**Results:**

Our initial search found a total of 2568 records and finally 29 trials were included in final analysis. Our results showed that adherence from VLCKD led to significant reduction in fasting blood sugar (WMD= -11.68 mg/dl; 95% CI: -18.79, -4.56; *P* = 0.001), HbA1c (WMD= -0.29; 95% CI: -0.44, -0.14; *P* < 0.001), HOMA-IR(WMD= -0.71; 95% CI: -1.14, -0.29; *P* = 0.001), insulin (WMD= -1.45; 95% CI: -2.54, -0.36; *P* = 0.009), triglyceride (WMD= -17.95; 95% CI: -26.82, -9.07; *P* < 0.001), systolic blood pressure (WMD= -2.85, 95% CI: -4.99, -0.71; *P* = 0.009) and diastolic blood pressure (WMD= -1.40; 95% CI: -2.66, -0.13; *P* = 0.03). We also found a significant increase in high-density lipoprotein (HDL) level after adherence from VLCKD diet (WMD = 3.93, 95% CI: 2.03, 5.84; *P* = 0.000). We couldn’t find any significant differences between groups in term of LDL and total cholesterol levels.

**Conclusion:**

People following a VLCKD experience a more significant improvement in cardiovascular risk factors when compared to individuals on control diets.

**Supplementary Information:**

The online version contains supplementary material available at 10.1186/s12986-024-00824-w.

## Introduction

T2DM represents a major global public health challenge, ranking as one of the most prevalent chronic illnesses, affecting millions of individuals worldwide [1]. In the United States, an estimated 37 million people, roughly equivalent to 10% of the population, are living with diabetes. Notably, T2DM accounts for approximately 90–95% of these cases [2]. The high and rising prevalence of T2DM in recent decades has spurred increased research efforts to investigate the efficacy of various interventions in managing this chronic condition [3–5]. In addition to impaired glycemic profile, patients with T2DM are at high risk for developing cardiovascular disease due to the presence of multiple risk factors, including high blood pressure, lipid profile disorders, and elevated inflammatory markers like CRP [6]. Consequently, a key focus for mitigating cardiovascular disease risk in diabetic patients lies in implementing effective lifestyle modifications, particularly those centered on dietary interventions [7–9].

Before the landmark discovery of exogenous insulin in the 1920s, management of diabetes mellitus relied heavily on strict dietary regimens that differed significantly from contemporary recommendations. These historical approaches often emphasized severe calorie restriction and limited carbohydrate intake, contrasting with the current focus on balanced, moderate-carbohydrate diets [10, 11]. For instance, a prominent example from 1923 involved a physician-recommended diabetic diet consisting of just 5% protein, 20% carbohydrate, and a remarkably high 75% fat [12, 13]. Clinical trials have shown that dietary interventions, including carbohydrate-restricted diets and low-glycemic index (GI) diets, can be effective in significantly lowering postprandial serum glucose concentrations [14–17]. While the degree of carbohydrate restriction varies across studies [18–20], a VLCKD is typically defined as a dietary regimen limiting carbohydrate intake to less than 50 g per day [21]. While some studies suggest potential benefits of low-carbohydrate diets for prediabetes and T2DM management, conflicting results and knowledge gaps prevent the establishment of a universally accepted optimal dietary approach [22, 23].

Diverging from a one-size-fits-all approach, both Diabetes UK and the American Diabetes Association (ADA) guidelines emphasize individualized dietary management for patients with diabetes. However, they achieve consensus on several core nutritional recommendations [24, 25]. In recent years, a growing body of research has investigated the impact of low-carbohydrate diets, particularly VLCKD, on cardiovascular risk in patients with T2DM. While some studies report positive effects on glycemic and lipid profiles [16], others reveal no significant advantage compared to other dietary approaches over the long term. This inconsistency highlights the need for further investigation to determine the optimal dietary strategy for managing T2DM and its associated cardiovascular risks [26]. Two recent systematic reviews and meta-analyses evaluated the effects of VLCKD in patients with T2DM and reported some beneficial outcomes [27, 28]. However, One meta-analysis included studies with varying degrees of carbohydrate restriction, not solely VLCKD [27]. Additionally, methodological shortcomings identified in the other study warrant reevaluation [28]. Moreover, the evidence’s degree of certainty has not been examined in any of the previous meta-analyses. Therefore, this study was conducted to assess the impact of a VLCKD diet on certain cardiovascular risk factors in patients with T2DM.

## Materials and methods

Following the principles of rigorous and transparent research methodology, we adhered to the Preferred Reporting Items for Systematic Reviews and Meta-Analysis (PRISMA) guidelines version 2020 [29], for conducting and reporting this study. Additionally, the protocol for this work was prospectively registered within the PROSPERO international prospective register of systematic reviews (Registration code: CRD42023475367).

### Search strategy

We conducted a comprehensive electronic database search to identify relevant studies, encompassing PubMed, Scopus, Google Scholar, Web of Science, Embase, and Cochrane Library. The search covered studies published from database inception up to March 2024 and employed the following search terms: “Diabetes Mellitus“[Title/Abstract] OR (“Noninsulin Dependent“[Title/Abstract] AND “Diabetes Mellitus“[Title/Abstract]) OR (“Diabetes Mellitus“[Title/Abstract] AND “Ketosis-Resistant“[Title/Abstract]) OR “Ketosis-Resistant Diabetes Mellitus“[Title/Abstract] OR (“Diabetes Mellitus“[Title/Abstract] AND “Non-Insulin-Dependent“[Title/Abstract]) OR “Non-Insulin-Dependent Diabetes Mellitus“[Title/Abstract] OR (“Diabetes Mellitus“[Title/Abstract] AND “stable“[Title/Abstract]) OR (“Diabetes Mellitus“[Title/Abstract] AND “Type II“[Title/Abstract]) OR “NIDDM“[Title/Abstract] OR “Type 2 Diabetes Mellitus“[Title/Abstract] OR “Noninsulin-Dependent Diabetes Mellitus“[Title/Abstract] OR “Type 2 Diabetes“[Title/Abstract] OR (“diabetes“[Title/Abstract] AND “Type 2“[Title/Abstract]) OR “Adult-Onset Diabetes Mellitus“[Title/Abstract] OR “Diabetes Mellitus“[Title/Abstract] OR (“Diabetes Mellitus“[Title/Abstract] AND “Insulin Dependent“[Title/Abstract]) OR “Insulin-Dependent Diabetes Mellitus“[Title/Abstract] OR “T2DM“[Title/Abstract]) AND (“ketogenic“[Title/Abstract] OR “very low carbohydrate” OR VLCKD OR “Ketogenic Diet“[Title/Abstract] OR (“diet“[Title/Abstract] AND “ketogenic“[Title/Abstract]) OR “keto diet“[Title/Abstract] OR “ketotic diet“[Title/Abstract]). To ensure comprehensive coverage, we not only searched electronic databases but also conducted a hand search of the reference lists from all studies included in the final analysis. Additionally, we searched grey literature sources to minimize the risk of overlooking relevant articles. Details of the specific search terms and strategies employed in each database are provided in the supplementary file.

### Eligibility criteria

Following the systematic search and transfer of records to Endnote software, two reviewers (MR and SAH) independently screened the studies. In the initial phase, they reviewed titles and abstracts to identify studies examining the impact of VLCKD on glycemic profile (FBS, insulin, HbA1C, HOMA-IR), lipid profile (triglyceride, total cholesterol, HDL, LDL), and blood pressure in patients with T2DM. Studies meeting these criteria were selected for inclusion. The second phase involved full-text review to ensure studies met all inclusion criteria: [1] investigating the effects of VLCKD diets on one of the mentioned biomarkers in T2DM patients, [2] being clinical trials, [3] having a carbohydrate intake less than 50 g/day, and [4] reporting mean or median with standard deviation (SD) or standard error (SE) or 95% CI for outcomes. We excluded studies that met the following criteria: [1] published in a language other than English or lacking a control group, [2] where no response was received from the corresponding author after two weeks of unsuccessful attempts to contact them via email, [3] review articles, case reports and case series, letters to the editor, conference abstracts, or one-arm clinical studies, [4] non-human trials. Regarding quality assessment between two researchers, the kappa statistic for agreement was 0.94. In instances of disagreement between the two reviewers during the initial or full-text screening phases, a third reviewer adjudicated the discrepancy.

### Data extraction

The following data were independently extracted by two researchers (MR and JH) from the articles included in the final analysis: author name, publication year, country, study design, participant numbers in intervention and control groups, age range, body weight, intervention duration, and study design (parallel/crossover). Additionally, post-intervention means and standard deviations (SD) of cardiovascular risk factors for both intervention and control groups, as well as their post-intervention mean (SD) changes, were extracted. If standard errors (SEs) or interquartile ranges were reported, they were converted to SDs for consistency.

### Assessment of the risk of bias and certainty of the evidence

To assess the risk of bias in studies with a randomized design, we employed the Cochrane Collaboration’s tool for assessing risk of bias [30]. This tool evaluates methodological aspects of studies that could potentially influence the results. Two researchers (MR and JH) independently evaluated the quality of each randomized study using seven domains according to the guidelines in the Cochrane Handbook for Systematic Reviews. Each domain was assessed using the terms “Low,” “High,” or “Unclear”. For non-randomized studies, we utilized the ROBINS-I (Risk Of Bias In Non-randomized Studies-of Interventions) tool [31]. This tool is specifically designed to evaluate the likelihood of bias in non-randomized studies comparing the health effects of different interventions.

We employed the GRADE (Grading of Recommendations Assessment, Development, and Evaluation) method to assess the certainty of the evidence [32]. The GRADE approach categorizes the certainty of evidence into four levels: “high,” “moderate,” “low,” and “very low.” Two reviewers (MR and RT) independently assessed the certainty of the evidence for each study using the GRADE criteria. These criteria consider potential biases arising from selection, participant performance, detection, attrition, and reporting across the included studies. Any disagreements between the reviewers were resolved by a third reviewer (JH).

### Statistical analysis

Effect sizes, representing the magnitude of treatment effects, were calculated as mean differences and standard deviations (SDs) for glycemic profiles (FBS, insulin, and HOMA-IR), lipid profiles (triglycerides, total cholesterol, HDL, and LDL cholesterol), and blood pressure (systolic and diastolic) levels between intervention and control groups. If studies reported data in other formats (standard errors [SEs] or interquartile ranges), these were converted to SDs for consistency. Standardized mean difference (SMD) and 95% confidence intervals (CIs) were used to present the effect sizes. Heterogeneity, which refers to the variability in effect sizes across studies, was assessed using both Cochran’s Q statistic and the I² statistic. A p-value greater than 0.10 for the Q-test or an I² statistic less than 50% suggests a lack of significant heterogeneity [33]. The random-effects model was employed when heterogeneity exceeded 50%, whereas the fixed-effects model was used for studies with low heterogeneity (I² below 50%). To explore potential sources of heterogeneity, we conducted subgroup analyses focusing on predefined variables such as duration of T2DM, baseline BMI, and intervention duration. To assess the influence of individual studies on the overall effect size, we conducted a sensitivity analysis using the one-study-omitted technique. Additionally, we visually inspected funnel plots to identify potential publication bias, where studies with statistically significant results are more likely to be published. Furthermore, Begg’s rank correlation and Egger’s linear regression tests were employed for a more robust evaluation of publication bias. All statistical analyses were performed using Stata software, version 14 SE (StataCorp, College Station, TX, USA). A p-value of less than 0.05 was considered statistically significant.

## Results

### Study selection

**Fig. 1:**
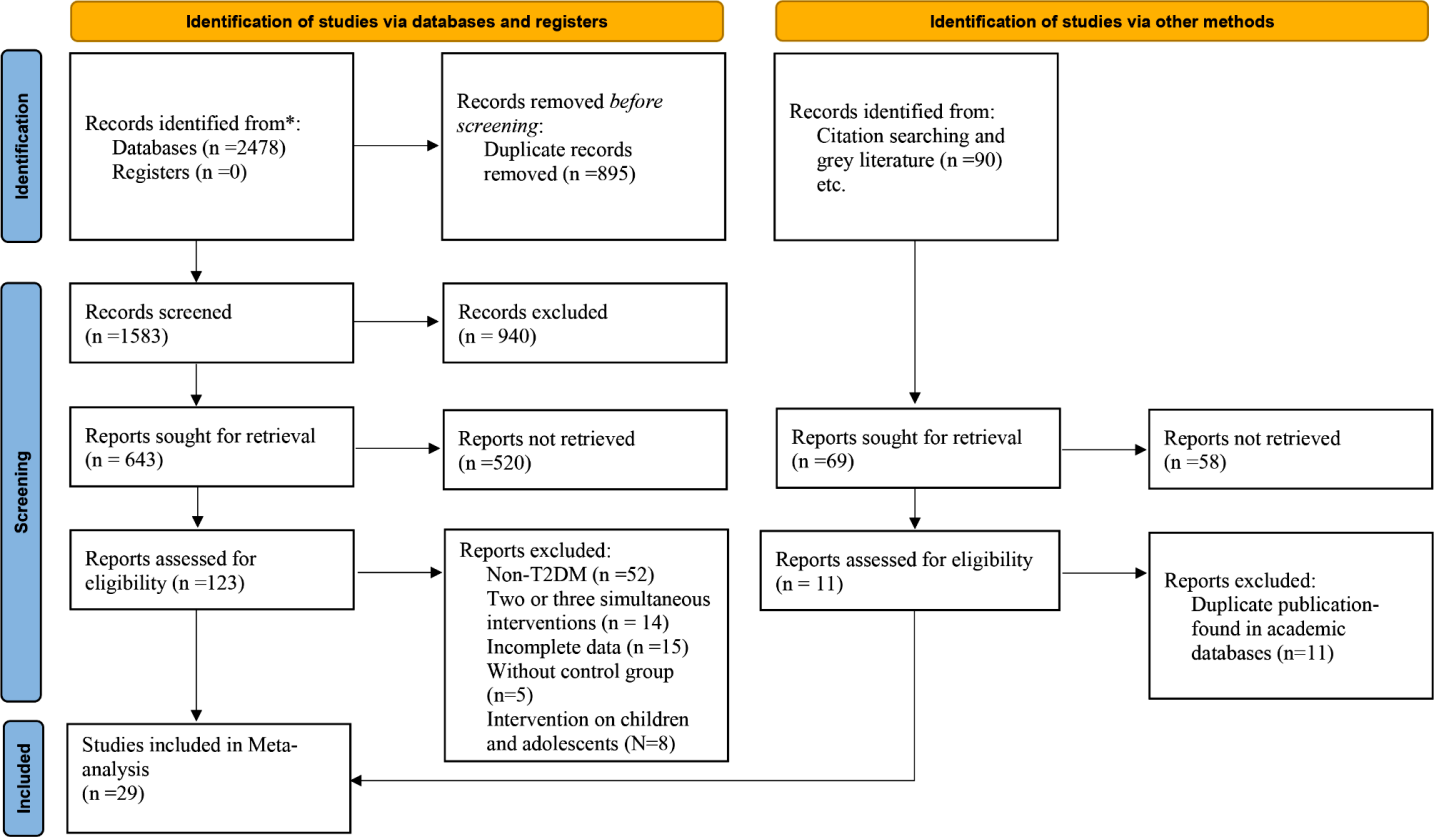
Preferred Reporting Items for Systematic Reviews and Meta-Analyses flow diagram (2020) of search process for studies examining the effects of Very Low Carbohydrate Ketogenic Diets on Cardiovascular Risk Factors among Patients with Type 2 Diabetes

Figure 1 illustrates the article filtering process. Our initial search identified a total of 2,568 records (2,478 from databases and 90 from grey literature. After deduplication using Endnote software, 1,583 studies proceeded to the first-stage screening based on titles and abstracts. One hundred and twenty-three articles advanced to the second phase involving full-text review. During this phase, we excluded studies that did not report our pre-defined outcomes, lacked a control group, involved interventions in children, adolescents, or non-T2DM/T1DM patients, included multiple simultaneous interventions, or were retrospective studies. Ultimately, 29 eligible clinical trials were included in the final analysis [22, 34–61].

### Characteristics of studies

Twenty-nine trials involving a total of 2,359 participants were included in the final analysis (Table 1). These studies were published between 2007 and 2023. Geographically, the studies originated from United States [22, 35, 36, 38, 40, 41, 43, 45, 47, 48, 61], Denmark [39, 55], Kuwait [46], Australia [34, 44, 50, 52], Spain [51], Canada [37, 60], Israel [42], Sweden [57, 58],UK [56], Germany [59], South Africa [54], Japan [49] and Italy [53]. The number of participants per study ranged from 11 to 349, with a mean participant age range of 18 to 68.7 years across the studies. Intervention durations varied from 4 days to 24 months. All studies included both men and women as participants.

**Table 1:** Characteristics of included studies

Study (Country)	Study Design	Intervention Duration	Intervention	Number of participants	Age (Mean ± SD)	Female, *N* (%)	BMI (Mean ± SD)	Duration of diabetes, years
[45](USA)	Randomized controlled trial	24-wk	VLCKD	21	51.2 ± 6.1	17 (79.3%)	37.9 ± 6.0	6.7 ± 3.12
			low-glycemic, reduced-calorie diet	29	50 ± 8.4	19 (67.3%)	37.8 ± 6.7	
[40] (USA)	Randomized controlled trial	3 months	VLCKD Low-fat diet	55 50	54 ± 6 53 ± 7	45(82%) 37 (74)	35 ± 6 37 ± 6	4.5 ± 3.12
[41](USA)	Randomized–controlled trial	24 months	VLCKD Low-fat diet	70 74	60.0 ± 8.9 60.0 ± 9.5	11 (15.7%) 4 (5.4%)	38.1 ± 5.5 36.9 ± 5.3	6.8 ± 3.4
[42](Israel)	Randomized–controlled trial	12 months	VLCKD ADA	26 26	57 ± 9 55 ± 8	13(50%) 14(53%)	33.1 ± 3.6 33.3 ± 3	5.8 ± 8.2
[46](Kuwait)	Randomized controlled trial	24-wk	VLCKD	78	45.1 ± 1.1	36.3 ± 0.5	36.3 ± 0.5	3.6 ± 1.5
			LCD	24	39.2 ± 0.7	40.0 ± 0.7	40.0 ± 0.7	
[47](USA)	Randomized controlled trial	48 -wk	VLCKD low-fat diet + orlistat	22 24	56.6 ± 7.3 54.7 ± 8.4	NR	38.3 ± 6.5 40.6 ± 6.4	5.9 ± 4.4
[47](Australia)	Randomized controlled trial	24 -wk	VLCKD high-unrefined carbohydrate, low fat diet (HC)	46 47	58 ± 6 7 58 ± 7	NR	34.2 ± 4.5 35.1 ± 4.1	6.5 ± 4
[34](Australia)	Randomized controlled trial	12 months	VLCKD	58	58.5 ± 1	49 (42%)	34.6 ± 0.4	7.5 ± 1.5
			Isocaloric High carbohydrate diet	57	58.4 ± 0.9			
[51] (Spain)	Open-label, multi-centric randomized clinical trial	4 months	VLCKD	45	54.89 ± 8.81	15 (33%)	33.25 ± 1.52	3.5 ± 1.7
			LCD	44	54.17 ± 7.97	16 (36%)	33.25 ± 1.60	
[35] (USA)	parallel-group randomized trial	12 months	LCKD	16	64.8 ± 7.7	9(56%)	35.9 ± 6.83	5.5 ± 2.5
			moderate-carbohydrate, calorie-restricted, low fat (MCCR) diet	18	55.1 ± 13.5	16(89%)	36.9 ± 6.53	
[43] (USA)	Parallel-group randomized trial	32 -wk	VLCKD	12	53 ± 10.2	6 (50%)	> 30	4.1 ± 5.7
			Plate method diet	13	58.2 ± 6.7	9 (69%)	> 30	
[37] (Canada)	Randomized crossover study	4 days	LCHF	11	64 ± 8	7 (63%)	> 30	6.4 ± 4.3
			Low-fat low-glycemic index	11				
[36] (USA)	Open label, non-randomized, controlled study	12 months	VLCKD	262	54 ± 8	172(66%)	40.4 ± 8.8	7 ± 1.5
			Regular diet	87	52 ± 8	50(58%)	36.7 ± 7.3	
[38](USA)	Open label, non-randomized, controlled study	24 months	VLCKD	194	53.8 ± 8.4	126(65%)	40.41 ± 8.42	8.44 ± 7.22
			Regular diet	68	51.4 ± 9.4	40(68%)	36.90 ± 7.41	
Moriconi et al. 2019(Italy)	clinical study	12 months	VLCKD	15	60.5 ± 10.2	7(47%)	39.5 ± 6.0	2.53 ± 1.19
			LCD	15	64.4 ± 8.8	7(47%)	32.2 ± 4.3	
[22](USA)	Randomized crossover trial	12 -wk	VLCKD	16	55.7 ± 8.40	7 (43.8%)	30.4 ± 5.2	6.5 ± 1.2
			Mediterranean diet	17	61.0 ± 10.5	6 (35.3)	31.0 ± 4.8	
[39](Denmark)	Open-label Randomized controlled trial	6 months	VLCD	49	57.3 ± 0.9	27 (55.1%)	32.5 ± 0.9	
			control diet	22	55.2 ± 2.7	13 (59.1%)	35.2 ± 1.4	5.2 ± 0.5
[50](Australia)	Randomized controlled trial	52 wk	VLCD High Carbohydrate	41 37	58 ± 7 58 ± 7	15(36%) 18(49%)	34.2 ± 4.5 35.1 ± 4.1	7 ± 5 9 ± 7
[61](USA)	Randomized controlled trial	4 months	VLCD DASH	23 25	60.09 ± 6.03 58.40 ± 8.11	15(65.21%) 16(64%)	35.1 ± 5.71 37.34 ± 6.20	6.8 ± 6.3 7.3 ± 5.9
Saslow et al.2013(USA)	Randomized controlled trial	3 months	VLCD Moderate carbohydrate	15 18	64.8 ± 7.7 55.1 ± 13.5	9(56.3%) 16(88.9%)	36.4 ± 5.8 35.7 ± 5.3	7.8 ± 7.5 6.4 ± 4.9
Hansen et al.2022(Denmark)	Randomized controlled trial	6 months	VLCD HCLF	110 55	57 ± 9 55 ± 12	62(56%) 34(62%)	33 ± 7 35 ± 8	4.5 ± 3.12 4.23 ± 2.9
[60](Canada)	Randomized controlled trial	6 months	VLCD LFD	60 61	65 ± 9 64 ± 10	29(48%) 35(57%)	33.2 ± 8.2 31.4 ± 6.2	10 ± 8 9 ± 6
[57](Sweden)	Randomized controlled trial	24 months	VLCD LFD	30 31	61.2 ± 9.5 62.7 ± 11	16(53.4%) 18(58%)	31.6 ± 5 33.8 ± 5.7	9.8 ± 5.5 8.8 ± 6.2
[49](Japan)	Randomized controlled trial	6 months	VLCD Low calorie diet	12 12	63.3 ± 13.5 63.2 ± 10.2	5(41.7%) 7(59%)	24.5 ± 4.3 27.0 ± 3.0	8.9 ± 3.6 9.5 ± 4.8
Tay et al.2017(Australia)	randomized controlled trial	2 years	VLCD High carbohydrate	33 28	58 ± 5.86 58 ± 5.39	12(36%) 14(50%)	34.2 ± 3.22 35.1 ± 2.96	6 ± 4.39 8 ± 5.40
[58](Sweden)	randomized controlled trial	6 months	VLCD Low fat diet	30 31	61 ± 9.5 63 ± 11	16(54%) 18(58%)	32 ± 5.1 34 ± 5.7	9.8 ± 5.5 8.8 ± 6.2
[56](UK)	randomized controlled trial	3 months	VLCD Healthy eating diet	12 14	55 ± 5 50 ± 12	10(83%) 9(64%)	35.1 ± 6.8 35.0 ± 7.4	5.5 ± 6.4 -
[59](Germany)	Randomized controlled trial	3 weeks	VLCD hypocaloric low fat	16 20	63 ± 8 62.5 ± 9	6(37%) 8(40%)	32.1 ± 4.5 30.8 ± 4.3	5.3 ± 2.6 5 ± 1.9
Breukelman et al. 2019(South Africa)	Randomized controlled trial	16 weeks	VLCD control group	10 13	54.2 ± 12.67 58.3 ± 5.53	6(60%) 9(69%)	38.9 ± 6.06 38.2 ± 10.66	7.36 ± 4.6 6.7 ± 5.1

VLCKD, very low-carbohydrate ketogenic diet; LFD, low-fat diet; LCD, Low-calorie diet; KD, ketogenic diet; VLCK, very low-calorie-ketogenic; LCHF, low-carbohydrate high-fat; ERD, Energy-Restricted Diabetes diet; VLCD, very low carbohydrate diet, NR, not reported

### Risk of bias

**Fig. 2:**
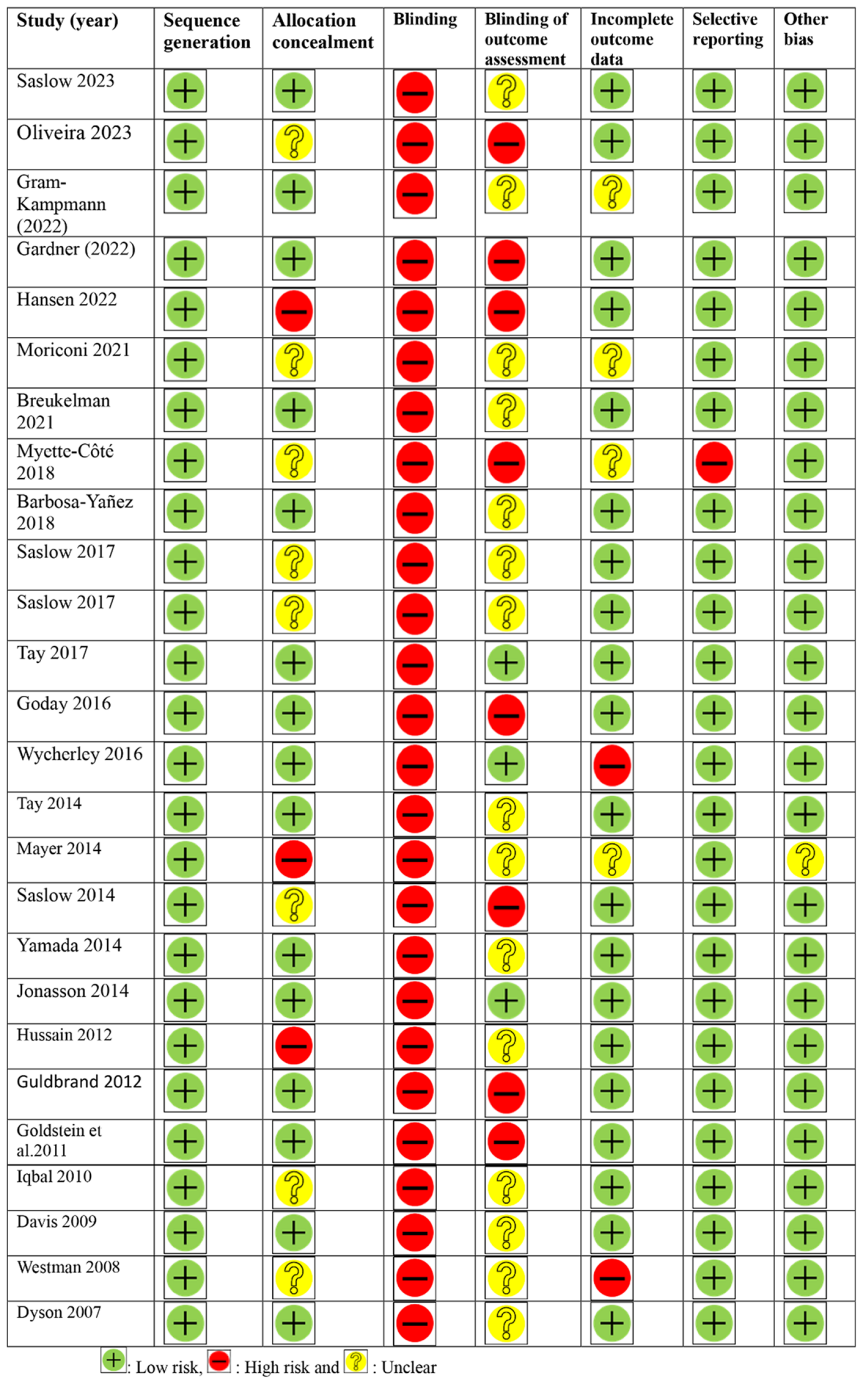
Study quality and risk of bias assessment of included studies in the meta-analysis

Risk of bias was assessed using the Cochrane Collaboration’s tool for randomized studies (Fig. 2) and the ROBINS-I tool for non-randomized studies (Table 2). In term of randomized studies, all studies used from acceptable random sequence generation, but fifteen trials, allocation concealment was acceptable [22, 34, 39, 40, 42, 44, 49, 51, 52, 54, 56–59, 61]. All the studies were prone to performance bias because participants were not blinded, and eight of them were susceptible to detection bias because outcome assessors were not blinded. Two studies had a high risk of attrition bias (due to dropouts), and one study had a high risk of reporting bias (due to selective reporting). In term of non-randomized studies, the overall quality for one study showed serious risk of bias and in two studies, there was a moderate risk of bias.

**Table 2:** Evaluation of risk of bias for each included study by the ROBINS-I tool

First author	Confounding	Selection of participants	Intervention	Deviations from intended interventions	Missing data	Measurement of outcomes	Selection of the reported result	Overall
Myette-Côté	Serious	Moderate	low	low	low	low	low	Serious
Bhanpuri	Moderate	low	low	low	low	low	low	Moderate
Athinarayanan	Moderate	low	low	low	low	low	low	Moderate

### Meta-analysis results

#### Effects of VLCKD on glycemic profile (FBS, insulin, HbA1c and HOMA-IR)

**Fig. 3:**
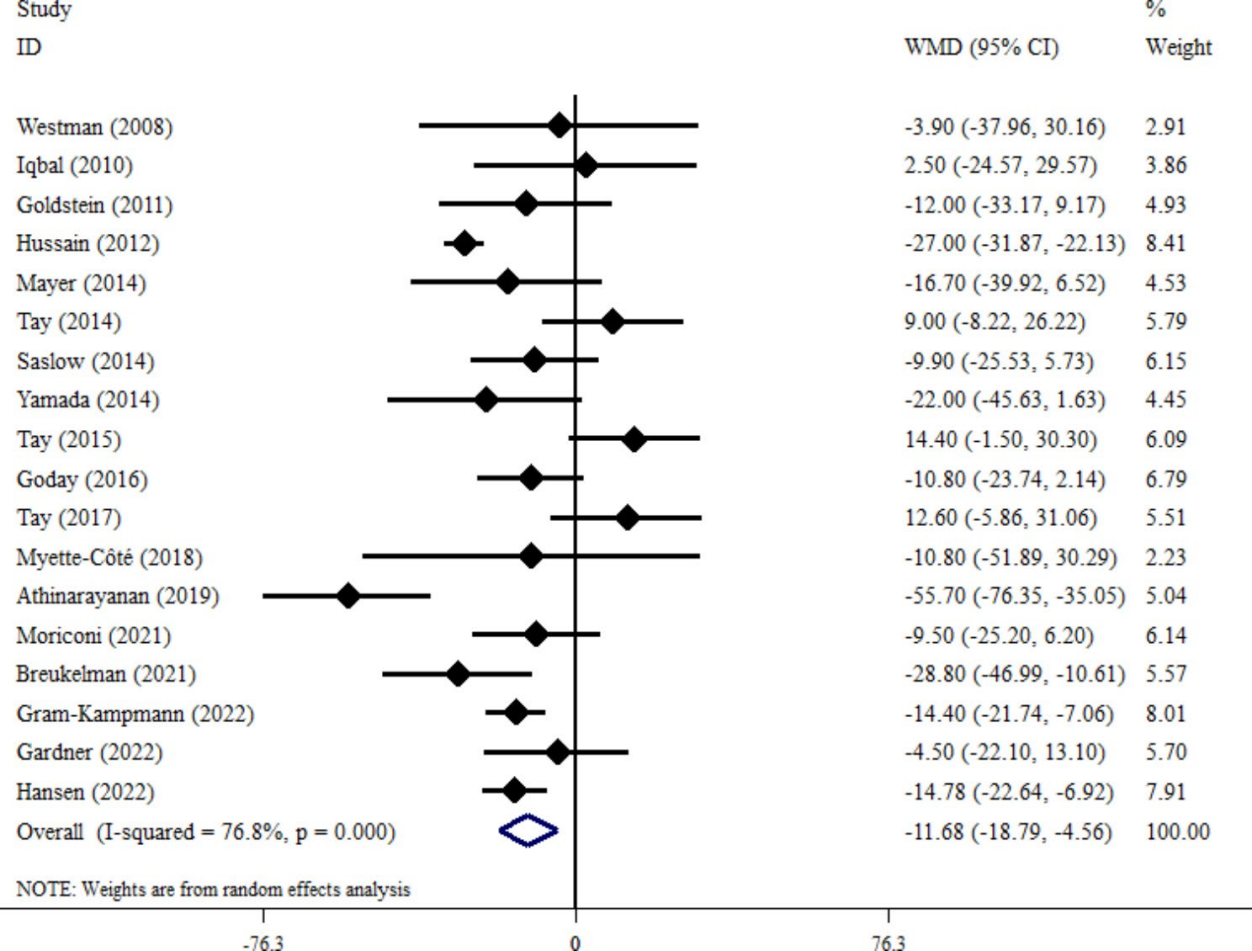
Forest plot detailing weighted mean difference and 95% confidence intervals for the impact of VLCKD on FBS level

**Fig. 4:**
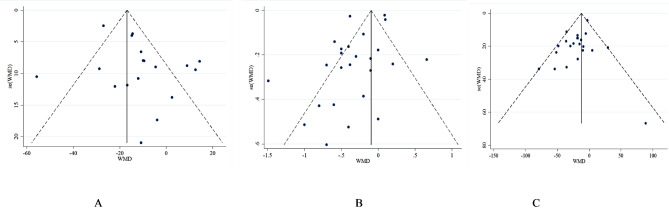
Funnel plots detailing publication bias in the studies selected for analysis. A, FBS; B, HbA1c; C, TG

The effect of VLCKD on FBS concentration was evaluated in 18 studies [22, 37–39, 41, 42, 44–55]. The combined analysis using the inverse variance method showed a significant decrease in FBS levels (WMD= -11.68 mg/dl; 95% CI: -18.79, -4.56; *P* = 0.001) (Fig. 3). Furthermore, a notable variation among studies was revealed, indicating significant between-study heterogeneity (I^2^ = 76.8%, *P* < 0.001). Subgroup analysis was conducted according to the length of intervention ( = < 6 months or > 6 months), T2DM duration (< 6 years or = > 6 years) and baseline BMI ( = < 35 or > 35 kg/m2) (Table 3). Subgroup analysis did not provide any explanation for between-study heterogeneity. The sensitivity analysis, where each trial was removed one at a time, indicated that no single trial significantly influenced the overall effect size. A funnel plot (Fig. 4A) indicated that there was no publication bias among the trials examining the impact of LCKD on FBS levels (Egger’s test *P* = 0.140; Begg’s test *P* = 0.0732) (See Fig. 4).

**Table 3:** Subgroup analyses for the effect of VLCKD on cardiovascular disease risk factors among the patients with T2DM

	Number of effect sizes	WMD (95% CI)	*P* effect	*P* within^1^	I^2^ (%)	*P* between^2^
**Subgroup analyses of VLCKD on FBS levels**						
Duration of follow up (months)						< 0.001
≤ 6	10	-13.66 (-21.74, -5.58)	0.001	< 0.001	69.5%	
> 6	8	-9.73 (-23.37, 3.90)	0.16	< 0.001	80.7%	
T2DM duration(years)						< 0.001
< 6	10	-19.14 (-22.37, -15.90)	< 0.001	0.001	54.5%	
>=6	8	-7.35 (-13.97, -0.74)	0.029	0.000	83.9%	
Baseline BMI (kg/m^2^)						< 0.001
=<35	10	-7.95 (-14.51, -1.38)	0.018	0.036	49.8%	
> 35	8	-17.09 (-30.34, -3.83)	0.012	0.000	79.6%	
**Subgroup analyses of VLCKD on HbA1C**						
Duration of follow up (months)						0.000
≤ 6	6	-0.28(-0.66, 0.09)	0.136	0.000	79.2%	
> 6	9	-0.23 (-0.42, -0.04)	0.02	0.000	85.5%	
T2DM duration(years)						0.000
< 6	11	-0.33 (-0.55, -0.11)	0.004	0.001	66.5%	
>=6	14	-0.23 (-0.38, -0.08)	0.003	0.000	81.4%	
Baseline BMI (kg/m^2^)						0.000
≤ 35	13	-0.18 (-0.38, 0.03)	0.09	0.000	80. 7%	
> 35	12	-0.43 (-0.67, -0.19)	0.000	0.000	90.6%	
**Subgroup analyses of VLCKD on TG**						
Duration of follow up (months)						0.000
≤ 6	12	-13.73(-24.29, -3.16)	0.011	0.216	23.2%	
> 6	10	-21.75 (-35.45, -8.05)	0.002	0.171	29.8%	
T2DM duration(years)						0.064
< 6	10	-14.13 (-23.76, -4.49)	0.004	0.265	19.4%	
>=6	12	-19.20 (-34.49, -3.91)	0.014	0.089	37.8%	
Baseline BMI (kg/m^2^)						0.000
≤ 35	10	-17.15 (-28.96, -5.34)	0.004	0.116	36.5%	
> 35	12	-19.08 (-33.13, -5.02)	0.008	0.133	32.2%	
**Subgroup analyses of VLCKD on TC**						
T2DM duration(years)						0.72
< 6	6	-1.06 (-12.6, 10.47)	0.85	0.001	77.3%	
>=6	6	-2.20 (-14.86, 10.47)	0.73	0.02	60.3%	
Duration of follow up (months)						0.83
≤ 6	7	-1.28 (-12.21, 9.65)	0.81	0.003	69.8%	
> 6	5	-2.07 (-15.87, 11.73)	0.77	0.005	72.9%	
Baseline BMI (kg/m^2^)						0.25
≤ 35	6	-3.90 (-14.18, 6.39)	0.45	0.024	61.5%	
> 35	6	1.98 (-12.48, 14.86)	0.86	0.001	68.3%	
**Subgroup analyses of VLCKD on LDL**						
T2DM duration(years)						< 0.001
< 6	9	5.75 (-0.29, 11.78)	0.06	0.006	62.7%	
>=6	13	2.32 (-5.29, 9.93)	0.55	0.000	76.1%	
Duration of follow up (months)						0.07
≤ 6	13	5.59 (1, 10.19)	0.017	0.065	40.3%	
> 6	9	2.02 (-7.71, 11.75)	0.68	0.000	84.4%	
Baseline BMI (kg/m^2^)						0.032
≤ 35	11	1.47 (-6.08, 9.02)	0.703	0.000	78.3%	
> 35	11	7.08 (0.93, 13.02)	0.024	0.000	60.1%	
**Subgroup analyses of VLCKD on HDL**						
T2DM duration(years)						< 0.001
< 6	11	4.74 (1.49, 7.99)	0.004	0.000	78.1%	
>=6	14	3.31 (0.87, 5.75)	0.008	0.000	72.6%	
Duration of follow up (months)						< 0.001
≤ 6	13	5.38 (2.42, 8.34)	0.000	0.000	70.7%	
> 6	12	2.62 (0.07, 5.17)	0.044	0.000	84%	
Baseline BMI (kg/m^2^)						0.321
≤ 35	12	2.45 (0.61, 4.29)	0.009	0.09	37.5%	
> 35	13	5.31 (2.36, 8.25)	0.001	0.000	80.1%	
**Subgroup analyses of VLCKD on SBP**						
T2DM duration(years)						0.56
< 6	7	-3.53 (-7.38, 0.32)	0.07	0.32	13.9%	
>=6	12	-2.62 (-5.25, 0.01)	0.047	0.007	57.4%	
Duration of follow up (months)						0.31
≤ 6	8	-2.52 (-4.94, -0.11)	0.041	0.55	0.0%	
> 6	11	-3.51 (-6.81, -0.2)	0.037	0.003	62.8%	
Baseline BMI (kg/m^2^)						0.63
≤ 35	9	-2.51 (-6.05, 1.03)	0.17	0.049	48.5%	
> 35	10	-3.15 (-5.94, -0.37)	0.026	0.052	42.8%	
**Subgroup analyses of LCKD on DBP**						
T2DM duration(years)						0.000
< 6	7	-2.73 (-5.75, 0.3)	0.078	0.001	74.5%	
>=6	11	-1.16 (-3.01, 0.69)	0.218	0.000	72.7%	
Duration of follow up (months)						0.000
≤ 6	7	-1.69 (-4.19, 0.8)	0.183	0.001	70.7%	
> 6	11	-1.66 (-3.69, 0.36)	0.107	0.000	74.3%	
Baseline BMI (kg/m^2^)						0.000
≤ 35	9	-2.41 (-5.12, 0.3)	0.081	0.000	81.8%	
> 35	9	-1.02(-2.77, 0.73)	0.254	0.02	55.9%	

^1^ P for heterogeneity, within subgroup

^2^ P for heterogeneity, between subgroups

**Fig. 5:**
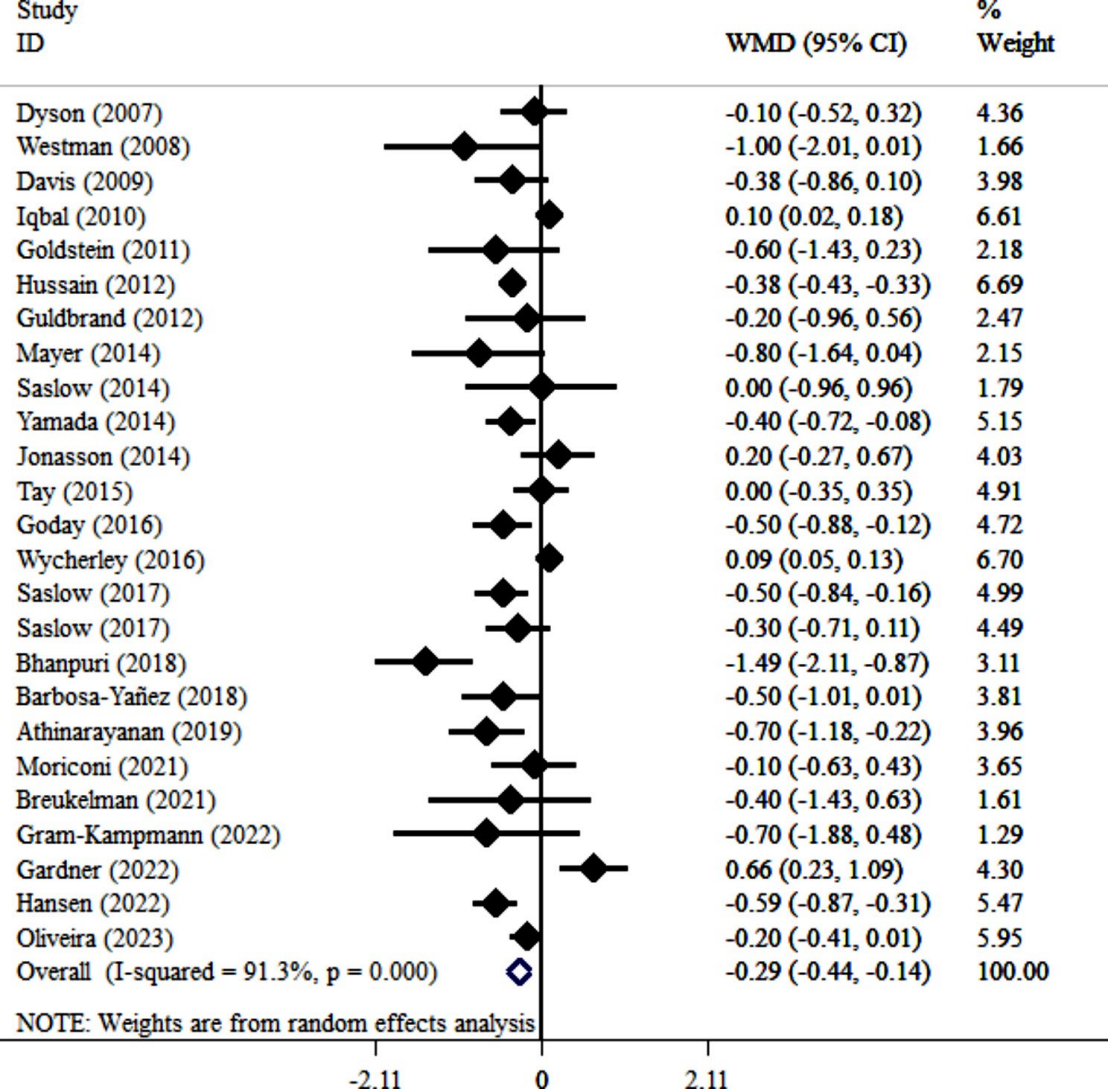
Forest plot detailing weighted mean difference and 95% confidence intervals for the impact of VLCKD on HbA1c level

Our meta-analysis of 25 clinical trials [22, 34–36, 38–43, 45–51, 53–60] demonstrated a significant reduction in HbA1c with VLCKD (WMD= -0.29; 95% CI: -0.44, -0.14; *P* < 0.001). However, substantial heterogeneity was present (I^2^ = 91.3%, *P* < 0.001) (Fig. 5). Subgroup analysis by baseline BMI identified it as a source of heterogeneity. VLCKD significantly reduced HbA1c only in patients with baseline BMI > 35 kg/m² (WMD=-0.43, -0.67, -0.19, *P* < 0.001). Sensitivity analysis confirmed the robustness of the overall effect size (CI range: -0.48, -0.09), indicating no single study unduly influenced the results. No publication bias was detected (Egger’s test *P* = 0.145; Begg’s test *P* = 0.834; Fig. 4B).

Initial analysis of eight clinical trials [38, 39, 44, 50–52, 55, 61] revealed a significant decrease in HOMA-IR index with VLCKD adherence (WMD= -0.71; 95% CI: -1.14, -0.29; *P* = 0.001) (Fig. 1S). However, significant heterogeneity was present (I^2^ = 83.6%, *P* < 0.001). Sensitivity analysis confirmed the robustness of the effect size. Also, no publication bias was detected (Egger’s test *P* = 0.083; Begg’s test *P* = 0.266).

Pool analysis using data from nine clinical trials [22, 37–39, 44, 45, 50, 52, 54] revealed a significant decrease in insulin concentration with VLCKD (WMD= -1.45; 95% CI: -2.54, -0.36; *P* = 0.009) (Fig. 2S). Notably, the analysis showed high homogeneity (I^2^ = 0.0%, *P* = 0.841), suggesting consistency across studies. Sensitivity analysis confirmed the robustness of the effect size, with no single trial significantly influencing the overall WMD. Furthermore, Begg’s test (*P* = 0.31) and Egger’s test (*P* = 0.38), alongside a visual inspection of the funnel plot, yielded no evidence of publication bias.

### Effects of VLCKD on lipid profile (TG, TC, LDL and HDL)

**Fig. 6:**
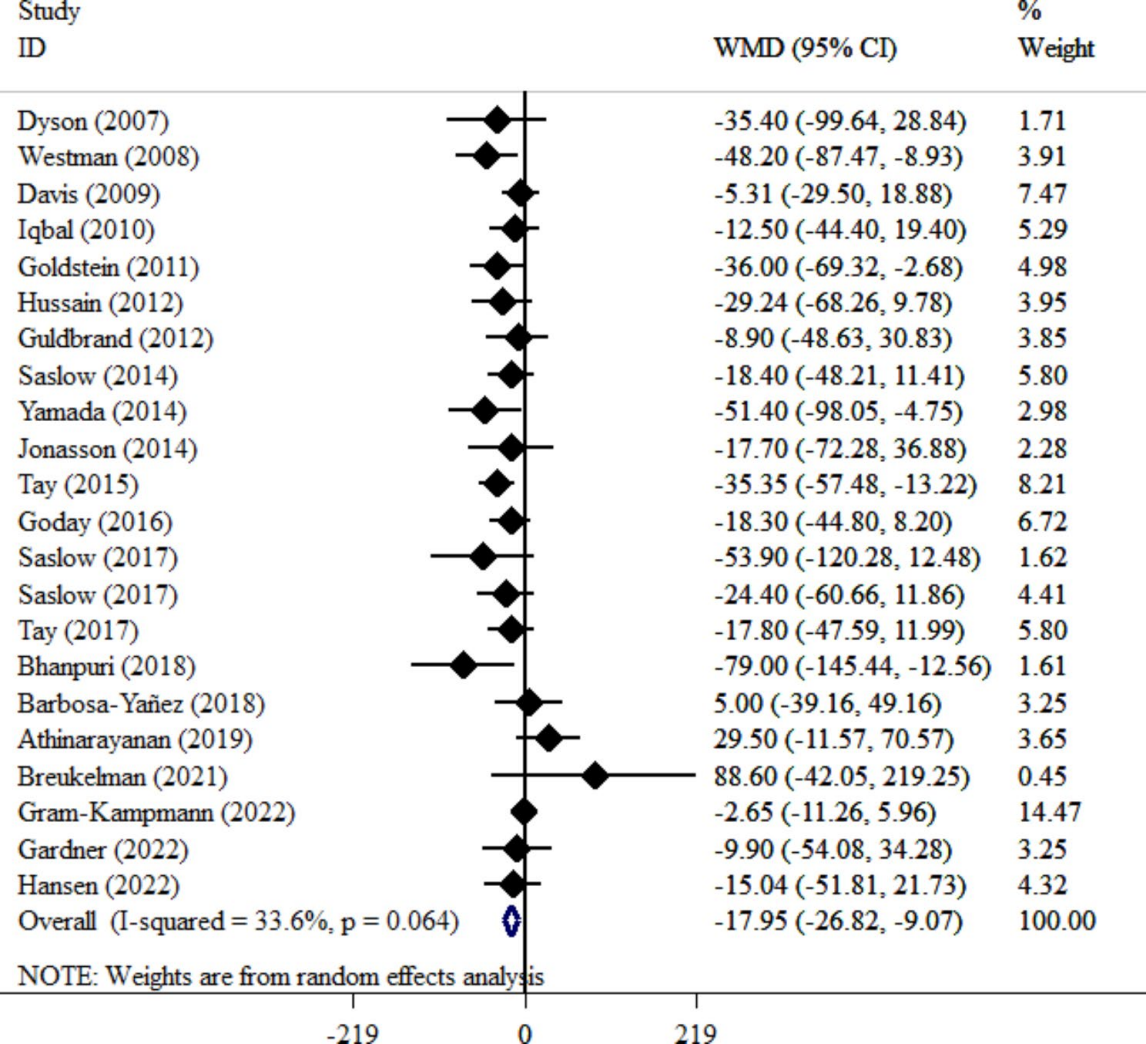
Forest plot detailing weighted mean difference and 95% confidence intervals for the impact of VLCKD on TG level

Our meta-analysis of 22 clinical trials [22, 35, 36, 38–43, 45, 46, 48–52, 54–59] demonstrated a significant reduction in TG levels with VLCKD adherence in patients with T2DM (WMD= -17.95; 95% CI: -26.82, -9.07; *P* < 0.001). Notably, heterogeneity was low and non-significant (I^2^ = 33.6%, *P* = 0.064) (Fig. 6), suggesting overall consistency across studies. VLCKD significantly decreased TG concentration in all evaluated subgroups, further supporting its efficacy. Leave-one-out sensitivity analysis confirmed the robustness of the effect size. Publication bias was unlikely based on Begg’s test (*P* = 0.612), Egger’s test (*P* = 0.069), and visual inspection of the funnel plot (Fig. 4C).

**Fig. 7:**
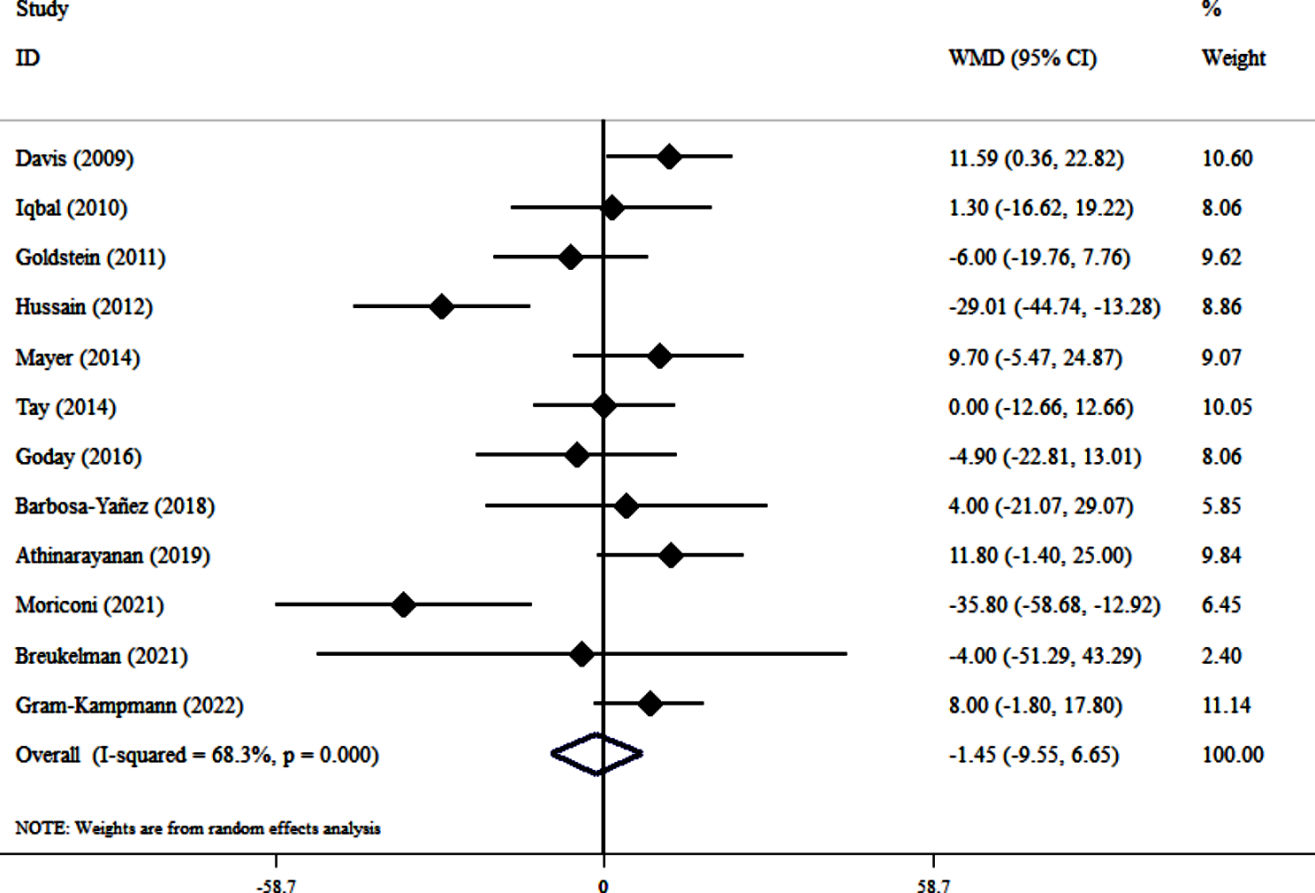
Forest plot detailing weighted mean difference and 95% confidence intervals for the impact of VLCKD on TC level

Pool analysis of 12 clinical trials [38–42, 44, 46, 47, 51, 53, 54, 59] revealed no significant effect of VLCKD on serum TC concentration (WMD= -1.45, 95% CI: -9.55, 6.65; *P* = 0.72). However, substantial heterogeneity was present (I^2^ = 68.3%, *P* < 0.001) (Fig. 7). Subgroup analysis failed to identify the source of this heterogeneity. Leave-one-out sensitivity analysis confirmed the robustness of the null effect. No publication bias was detected (Egger’s test, *P* = 0.154; Begg’s test, *P* = 0.14) (Fig. 3S).

Pool analysis of 22 trials [22, 35, 36, 38–43, 45, 46, 48–52, 54–59] revealed a non-significant increase in LDL concentration with VLCKD (WMD = 4.21, 95% CI: -0.54, 8.96; *P* = 0.08) (Fig. 4S). However, substantial heterogeneity was present (I², 71%; *P* < 0.001). Subgroup analysis identified potential moderators of this effect. In studies with intervention ≤ 6 months (WMD, 5.59 mg/dL; 95% CI, 1.00 to 10.19; *P* = 0.017) and baseline BMI > 35 kg/m² (WMD, 7.08 mg/dL; 95% CI, 0.93 to 13.02; *P* = 0.024), VLCKD significantly increased LDL concentration. Leave-one-out sensitivity analysis confirmed the robustness of the overall effect size. No publication bias was detected (Begg’s test, *P* = 0.86; Egger’s test, *P* = 0.214).

A meta-analysis of 25 trials [22, 35, 36, 38–43, 45, 46, 48–52, 54–59] revealed a significant increase in HDL-C concentration with VLCKD adherence in patients with T2DM (WMD = 3.93, 95% CI: 2.03, 5.84; *P* = 0.000) (Fig. 5S). However, substantial heterogeneity was present (I², 74.2%; *P* < 0.001). Subgroup analysis suggested that the intervention’s effectiveness may be modified by baseline characteristics and duration. Specifically, significant improvements in HDL-C were observed in participants with baseline BMI ≤ 35 kg/m², T2DM duration ≥ 6 years, and intervention time exceeding 6 months. Leave-one-out sensitivity analysis confirmed the robustness of the pooled effect size. Publication bias was unlikely (Egger’s test, *P* = 0.359; Begg’s test, *P* = 0.83).

### Effects of VLCKD on blood pressure

**Fig. 8:**
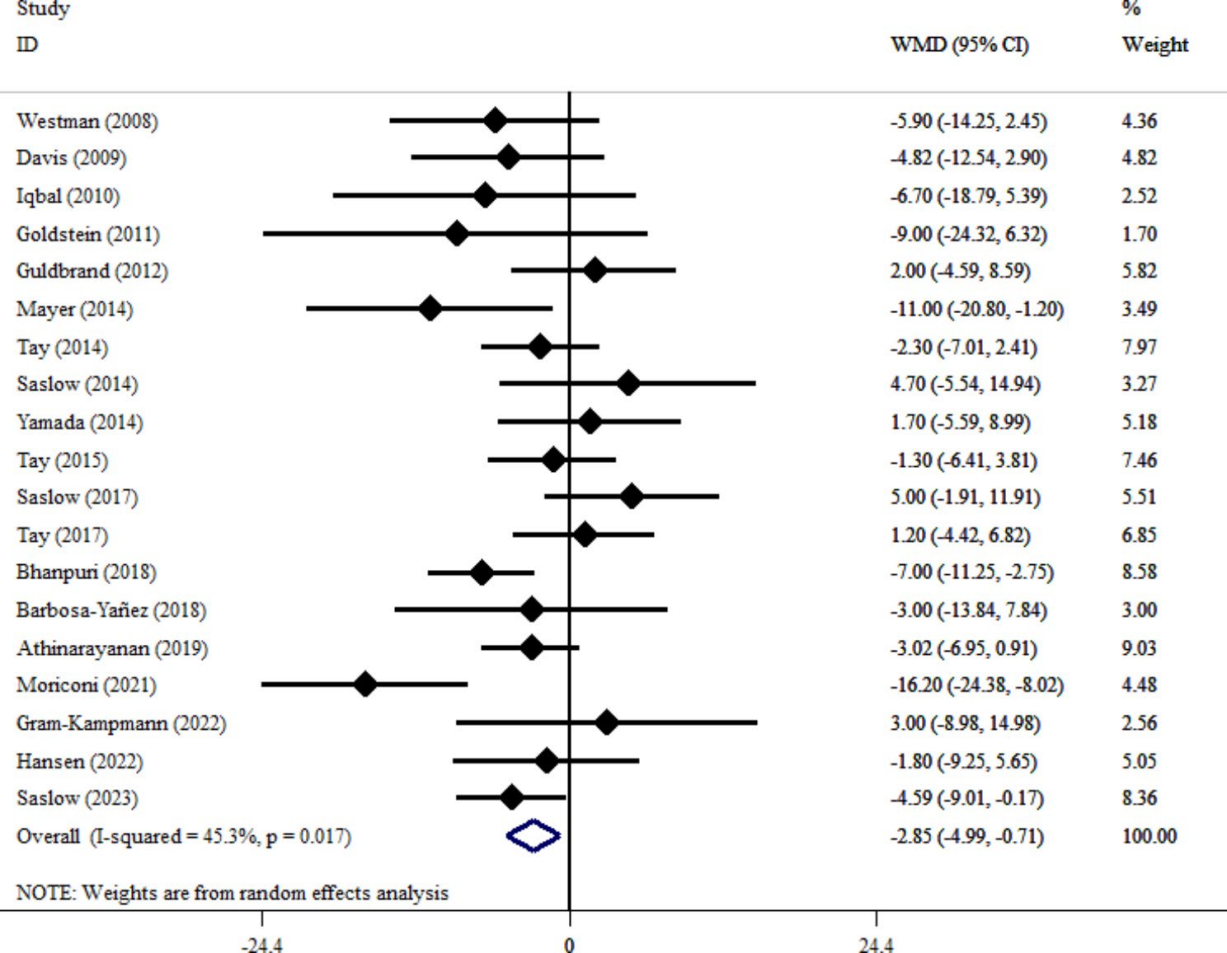
Forest plot detailing weighted mean difference and 95% confidence intervals for the impact of VLCKD on SBP

VLCKD adherence significantly reduced SBP in patients with T2DM [35, 36, 38–42, 44, 45, 47–50, 53, 55, 57, 59, 61] (WMD= -2.85, 95% CI: -4.99, -0.71; *P* = 0.009) (Fig. 8). Moderate heterogeneity was present (I², 45.3%; *P* = 0.017). When it comes to trials related to SBP, there was no evidence of publication bias found in both Begg’s test (*P* = 0.42) and Egger’s test (*P* = 0.99), as well as in the visual examination of the funnel plot. Subgroup analysis revealed a significant SBP reduction with VLCKD in participants with baseline BMI > 35 kg/m² (WMD, -3.15 mmHg; 95% CI, -5.94 to -0.37; *P* = 0.026), and the effect did not differ by follow-up duration. Leave-one-out sensitivity analysis confirmed the robustness of the effect size.

Strict adherence to a VLCKD resulted in a significant reduction in diastolic blood pressure (DBP) (WMD= -1.40; 95% CI: -2.66, -0.13; *P* = 0.03) (Fig. 6S) in an analysis of 18 clinical trial arms [35, 36, 38–42, 44, 45, 47–50, 53, 55, 57, 59, 61]. However, substantial heterogeneity was present among studies (I^2^ = 78.2%, *P* < 0.001). Subgroup analysis could not identify the source of heterogeneity. The leave-one-out sensitivity analysis showed that leaving each of trials had no significant effect on the pooled effect size. A funnel plot demonstrated no publication bias of trials in investigating the effect of VLCKD on DBP level (Egger’s test *P* = 0.44; Begg’s test *P* = 0.17).

### Grading of evidence

The GRADE approach was used to evaluate the certainty (quality) of evidence. Triglyceride levels received a moderate-certainty rating (Table 4). The certainty of evidence for FBS and TC was downgraded to low. Evidence for HbA1c, SBP, and LDL-C received very low certainty ratings.

**Table 4:** GRADE profile of very low Carbohydrate Ketogenic diets on Cardiovascular Risk factors in adults

	Quality assessment					Summary of findings			
Outcomes	Risk of bias	Inconsistency	Indirectness	Imprecision	Publication bias	Number of Intervention/Control	WMD (95%CI)	Heterogeneity (I^2^)	Quality of evidence
FBS	serious limitations	serious Limitations	No serious limitations	No serious limitations	No serious limitations	860/610	-13(-21.62, -4.38)	77.7%	⊗⊗ΟΟ Low
HbA1c	serious limitations	Very serious Limitations	No serious limitations	serious limitations	No serious limitations	1320/856	-0.28 (-0.47, -0.08)	94.5	⊗ΟΟΟ Very Low
TG	serious limitations	Low serious Limitations	No serious limitations	No serious limitations	No serious limitations	1210/795	-17.11(-29.46, -4.76)	45.3%	⊗⊗⊗Ο Moderate
TC	serious limitations	serious Limitations	No serious limitations	No serious limitations	No serious limitations	840/633	-1.85 (-10.69, 6.99)	74%	⊗⊗ΟΟ Low
SBP	serious limitations	serious Limitations	No serious limitations	serious Limitations	No serious limitations	1135/769	-4.85(-8.04, -1.65)	53.7%	⊗ΟΟΟ Very Low
LDL	serious limitations	Very serious limitations	serious limitations	No Limitations	No serious limitations	1198/812	-4.84 (-11.85, 2.17)	92%	⊗ΟΟΟ Very Low

FBS, Fasting blood sugar; HbA1c, hemoglobin A1c; LDL, low density lipoprotein; SBP, systolic blood pressure; TC, total cholesterol; TG, triglyceride

## Discussion

This systematic review and meta-analysis investigated the effects of VLCKDs on cardiovascular risk factors in patients with T2DM. Our findings demonstrated that adherence to a VLCKD significantly improved glycemic control, as evidenced by reductions in FBS, HbA1c, and insulin levels. Additionally, VLCKDs significantly lowered triglyceride levels and increased HDL-cholesterol levels, both of which are favorable for cardiovascular health. However, we observed no significant changes in TC or LDL-cholesterol levels.

Patients with T2DM exhibit a markedly elevated prevalence of CVD. Indeed, a significant proportion of individuals diagnosed with T2DM develop CVD within a few years. This underscores the critical importance of prioritizing CVD risk factor management in this patient population. Our study investigated the effects of adherence to VLCKD, known to induce ketosis, on serum levels of FBS and HbA1c. Consistent with previous research, we observed reductions in these markers. Additionally, existing studies suggest that VLCKD can improve blood sugar control, promote weight loss, and decrease the risk of metabolic syndrome [62]. Dietary carbohydrates significantly impact postprandial glycemia, promoting fluctuations in blood glucose levels and potentially inducing hyperinsulinemia. Consequently, carbohydrate restriction has been shown to be an effective strategy for enhancing glycemic control, lowering insulin demand, and improving insulin sensitivity [63–65]. In addition to limiting the consumption of carbohydrates, adherence to the VLCKD triggers a condition known as nutritional ketosis in individuals [66, 67]. The metabolic consequences associated with nutritional ketosis, such as the reduction of fat reserves and diminished appetite, could potentially be linked to the advantageous outcomes observed with the VLCKD [68–70]. Diminished hunger sensations could have contributed to a decrease in energy intake among participants following the VLCKD. Nevertheless, it is essential to highlight that in most of the studies incorporated in this analysis, the control diet group adhered to an energy-restricted regimen of 500 kcal/d, whereas the VLCKD group had unrestricted access to food.

Several studies have established the beneficial effects of weight-loss-focused nutritional interventions on T2DM management [45]. Supporting this notion, recent American Diabetes Association (ADA) guidelines recommend a 5–10% body weight reduction within six months to significantly improve T2DM symptoms and disease control [25]. Notably, VLCKDs have demonstrated promising weight loss effects in numerous studies. A meta-analysis by Bueno et al. revealed that VLCKD adherence led to significant long-term weight loss [19]. These findings are corroborated by additional meta-analyses [71, 72]. VLCKDs promote satiety by encouraging higher intakes of fat and protein. These macronutrients exhibit a slower rate of gastric emptying compared to carbohydrates, leading to sustained feelings of fullness [69, 73]. Additionally, VLCKDs induce ketosis, a metabolic state where the body produces ketones that further suppress appetite. This enhanced satiety may contribute to weight loss, particularly the reduction of visceral and abdominal fat, which is known to play a crucial role in managing T2DM according to past research findings [74].

The enhanced glycemic control observed with the VLCKD could potentially be linked to the greater magnitude of weight reduction experienced by individuals adhering to this dietary approach [75, 76]. Our study revealed a significant decrease in HbA1c solely within the subgroup of patients with a body BMI exceeding 35 kg/m2 adhering to a VLCKD. This observation might be explained by the greater reduction in adipose tissue stores that occurs during nutritional ketosis in individuals with a higher BMI [68, 77, 78]. Intriguingly, some studies suggest that ketogenic diets can improve glucose metabolism independent of weight loss. Gumbiner et al. [79] examined the impact of ketosis and ensuing weight reduction on glycemic regulation among individuals following ketogenic dietary patterns. Their study revealed a notable enhancement in glycemic parameters among those adhering to the ketogenic diet compared to the nonketogenic counterpart, despite comparable levels of weight loss achieved by participants in both groups. Given its low-glycemic nature, VLCKD might directly ameliorate glycemic management by concurrently fostering weight reduction [80, 81]. Our study observed a reduction in HbA1c levels in patients with T2DM adhering to a VLCKD. This aligns with findings from Elhayany et al. [82]. where a low-carbohydrate Mediterranean diet also led to significant HbA1c reductions. Similarly, a systematic review by Sainsbury et al. demonstrated the greater efficacy of low-carbohydrate diets compared to moderate or high-carbohydrate diets in lowering HbA1c levels [71]. Notably, previous studies have shown that each 1% reduction in HbA1c is associated with a reduction in the risk of cardiac infarction by 14% and micro-vascular complications by 37% [83–85]. In our analysis, trials reported HbA1c reductions ranging from 0.1 to 1.45% following VLCKD intervention. The duration of the intervention is likely a contributing factor to the variation in HbA1c reductions observed across studies.

Our study observed a significant decrease in serum TG and an increase in HDL cholesterol in patients adhering to a VLCKD. Notably, no significant changes were observed in LDL cholesterol or TC. Consistent with our findings, a previous meta-analysis reported a significant reduction in TG concentration following a VLCKD [71, 86, 87]. Dietary carbohydrate restriction, particularly of simple carbohydrates, has been demonstrated in prior studies to be more effective in reducing triglycerides compared to dietary fat restriction. This effect is likely mediated, in part, by a reduction in postprandial insulin secretion. As an anabolic hormone, insulin promotes both lipogenesis and the conversion of glucose to fatty acids [84, 88]. Our study observed a significant increase in HDL-C concentration within the VLCKD group. This finding aligns with the observations reported by Volek et al., who proposed that the preservation of HDL-C levels and reduced postprandial lipemia in individuals adhering to a VLCKD may be attributable to lowered triacylglycerol (TAG) levels [89]. Typically, VLCKD are characterized by a significant proportion of dietary fat. The replacement of carbohydrates with fats in these diets may have contributed to a deceleration in the reduction of serum LDL-C levels. The results of a meta-analysis study, which assessed the impact of a ketogenic diet versus a low-fat diet among non-diabetic individuals with obesity, observed that serum LDL-C levels were elevated in the VLCKD group compared to the low-fat diet group [19]. Ketogenic dietary patterns elevate serum LDL-C levels due to their heightened saturated fat content. The augmented intake of fat and restriction of carbohydrates foster the proliferation of large LDL-C particles, which are deemed to be less atherogenic. Consequently, juxtaposing the elevated LDL-C levels observed with ketogenic diets against those induced by alternative dietary regimens may not be entirely appropriate [90, 91].

Our study revealed a noteworthy reduction in both SBP and DBP among individuals with T2DM who adhered to a VLCKD. Consistent with our findings, certain studies propose that the substitution of carbohydrates with proteins and monounsaturated fats may confer an additional reduction in blood pressure, surpassing the anticipated decline solely from sodium restriction [92]. Furthermore, reports indicate that a low carbohydrate diet can enhance arterial function and trigger the phosphorylation of endothelial nitric oxide synthase (eNOS) enzyme, a critical factor for vascular vasodilation [93, 94]. Various other mechanisms have been proposed to explain these favorable outcomes, including heightened relaxation of mesenteric arteries attributed to enhanced endothelium-dependent response (acetylcholine), coupled with diminished contraction (potassium chloride, phenylephrine), and elevated phosphorylation of eNOS^Ser1177^ in arteries [95–97].

This study exhibited several strengths compared previous meta-analysis, including a comprehensive search strategy, appropriate statistical methods, sub group analysis based on different variables and a thorough evaluation of the quality and certainty of the evidence. The use of the GRADE approach to assess the certainty of evidence adds credibility to the findings by systematically evaluating the quality of evidence across outcomes, helping readers understand the reliability of the results. However, several limitations were identified in the present meta-analysis. Firstly, in terms of study design, the main issue in terms of internal validity of these studies is the lack of blinding of participants and study personnel, something that is difficult to achieve given such an intervention; however, this should be acknowledged when interpreting results. Secondly, it relied on aggregated data from the included studies rather than individual patient data. Thirdly, the heterogeneity among the studies may restrict the generalizability of the findings, although subgroup analyses were conducted to address confounding variables. Fourthly, some studies compared two different types of diets rather than employing a routine diet for the control group, which could introduce bias. Additionally, the short duration of most included studies hinders the assessment of long-term cardiovascular risks associated with VLCKDs in patients with T2DM. Moreover, the certainty of evidence was generally low for most outcomes. Finally, some studies reported low adherence to the VLCKD, highlighting challenges in maintaining adherence and sustainability over the long term. It’s important to recognize that strict adherence to VLCKDs may be challenging for some individuals, and the potential long-term risks and benefits should be carefully weighed.

## Conclusion

In conclusion, the results of the present study showed that adherence from a VLCKD diet among the patients with T2DM can act as an effective nutritional approach in improving the glycemic profile and reducing the risk of cardiovascular complications. The observed improvements in glycemic control, as evidenced by reductions in FBS levels, glycated HbA1c, and insulin levels, are of paramount importance in the management of T2DM. These improvements not only contribute to better disease control but also hold promise for reducing the risk of microvascular and macrovascular complications associated with poorly managed diabetes. Moreover, the significant reductions in TG levels and increases in HDL-C levels are particularly noteworthy from a cardiovascular risk perspective. Elevated TG levels are a well-established risk factor for CVD, while higher HDL-C levels are associated with a reduced risk of CVD events. Therefore, the observed changes suggest a potential cardioprotective effect of VLCKDs in patients with T2DM. However, further research is warranted to elucidate the long-term effects and sustainability of VLCKDs in managing T2DM and reducing cardiovascular risk. Healthcare providers should consider individual patient preferences and characteristics when recommending dietary interventions, and close monitoring is necessary to ensure safety and efficacy.

### Electronic supplementary material

Below is the link to the electronic supplementary material.

Supplementary Material 1

Supplementary Material 2

## Data Availability

The original contributions presented in the study are included in the article, further inquiries can be directed to the corresponding author/s.
