# Supplementary material for: Impact of very low carbohydrate ketogenic diets on cardiovascular risk factors among patients with type 2 diabetes; GRADE-assessed systematic review and meta-analysis of clinical trials
Source: Nutr Metab (Lond). 2024 Jul 19;21:50. doi: 10.1186/s12986-024-00824-w (PMC11264514; doi:10.1186/s12986-024-00824-w)
Supplement: Supplementary file 2 — Supplementary Material 2 [file 12986_2024_824_MOESM2_ESM.docx]

**Online Supplementary Information**

**Impact of** **Very Low Carbohydrate Ketogenic Diets on Cardiovascular Risk Factors among Patients with Type 2 Diabetes; GRADE-assessed Systematic Review and Meta-Analysis of Clinical Trials**

Parisa Ghasemi^1^, Malihe Jafari^2^, Saber Jafari Maskouni^3^, Seyed Ahmad Hosseini^4^, Roksaneh Amiri^5^, Jalal Hejazi^6^, Mahla Chambari^7^, Ronia Tavasolian^6^, Mehran Rahimlou^6*^

1. Research Committee, Medical School, Arak University of Medical Sciences, Arak, Iran
2. Department of Exercise physiology, Faculty of Physical Education and Sports Sciences, Allameh Tabataba'i University, Tehran, Iran
3. Department of nutrition, School of Public health, Jiroft University of Medical Sciences, Jiroft, Iran
4. Department of Nutrition, School of Allied Medical Sciences, Ahvaz Jundishapur University of Medical Sciences, Ahvaz, Iran
5. Mofid Childrens Hospital Clinical Research Development Unit, Shahid Beheshti University of Medical Science, Tehran, Iran
6. Department of Nutrition, School of Public Health, Zanjan University of Medical Sciences, Zanjan, Iran
7. Noncommunicable Diseases Research Center, Neyshabur University of Medical Sciences, Neyshabur, Iran

*Correspondence to: Mehran Rahimlou

Mehran Rahimlou, PhD, Central Office of Zanjan University of Medical Sciences, Azadi Sq. Zanjan, Iran. Tel: 0098-24-33420651, Email: [Rahimlum@gmail.com](mailto:Rahimlum@gmail.com)

**Running Title:** Very Low Carbohydrate Ketogenic Diets and Diabetes

**Search Strategies**

1. Search strategy in Pubmed

("Diabetes Mellitus"[Title/Abstract] OR ("Noninsulin-Dependent"[Title/Abstract] AND "Diabetes Mellitus"[Title/Abstract]) OR ("Diabetes Mellitus"[Title/Abstract] AND "Ketosis-Resistant"[Title/Abstract]) OR "Ketosis-Resistant Diabetes Mellitus"[Title/Abstract] OR ("Diabetes Mellitus"[Title/Abstract] AND "Non-Insulin-Dependent"[Title/Abstract]) OR "Non-Insulin-Dependent Diabetes Mellitus"[Title/Abstract] OR ("Diabetes Mellitus"[Title/Abstract] AND "stable"[Title/Abstract]) OR ("Diabetes Mellitus"[Title/Abstract] AND "Type II"[Title/Abstract]) OR "NIDDM"[Title/Abstract] OR "Type 2 Diabetes Mellitus"[Title/Abstract] OR "Noninsulin-Dependent Diabetes Mellitus"[Title/Abstract] OR "Type 2 Diabetes"[Title/Abstract] OR ("diabetes"[Title/Abstract] AND "Type 2"[Title/Abstract]) OR "Adult-Onset Diabetes Mellitus"[Title/Abstract] OR "Diabetes Mellitus"[Title/Abstract] OR ("Diabetes Mellitus"[Title/Abstract] AND "Insulin Dependent"[Title/Abstract]) OR "Insulin-Dependent Diabetes Mellitus"[Title/Abstract] OR "T2DM"[Title/Abstract]) AND ("ketogenic"[Title/Abstract] OR "Ketogenic Diet"[Title/Abstract] OR ("diet"[Title/Abstract] AND "ketogenic"[Title/Abstract]) OR "keto diet"[Title/Abstract] OR "ketotic diet"[Title/Abstract])

1. Search strategy in Scopus

( ( TITLE-ABS-KEY ( "Diabetes Mellitus" ) OR TITLE-ABS-KEY ( ( "Noninsulin-Dependent" AND "Diabetes Mellitus" ) ) OR TITLE-ABS-KEY ( ( "Diabetes Mellitus" AND "Ketosis-Resistant" ) ) OR TITLE-ABS-KEY ( "Ketosis-Resistant Diabetes Mellitus" ) OR TITLE-ABS-KEY ( ( "Diabetes Mellitus" AND "Non-Insulin-Dependent" ) ) OR TITLE-ABS-KEY ( "Non-Insulin-Dependent Diabetes Mellitus" ) OR TITLE-ABS-KEY ( ( "Diabetes Mellitus" AND stable ) ) OR TITLE-ABS-KEY ( ( "Diabetes Mellitus" AND "Type II" ) ) OR TITLE-ABS-KEY ( niddm ) OR TITLE-ABS-KEY ( "Type 2 Diabetes Mellitus" ) OR TITLE-ABS-KEY ( "Noninsulin-Dependent Diabetes Mellitus" ) OR TITLE-ABS-KEY ( "Type 2 Diabetes" ) OR TITLE-ABS-KEY ( ( diabetes AND "Type 2" ) ) OR TITLE-ABS-KEY ( "Adult-Onset Diabetes Mellitus" ) OR TITLE-ABS-KEY ( ( "Diabetes Mellitus" AND "Insulin-Dependent" ) ) OR TITLE-ABS-KEY ( ( "Diabetes Mellitus" AND "Insulin Dependent" ) ) OR TITLE-ABS-KEY ( ( "Insulin-Dependent Diabetes Mellitus" ) ) OR TITLE-ABS-KEY ( iddm ) OR TITLE-ABS-KEY ( t2dm ) ) ) AND ( ( TITLE-ABS-KEY ( ketogenic ) OR TITLE-ABS-KEY ( "Ketogenic Diet" ) OR TITLE-ABS-KEY ( ( diet AND ketogenic ) ) OR TITLE-ABS-KEY ( "keto diet" ) OR TITLE-ABS-KEY ( "ketogenous diet" ) OR TITLE-ABS-KEY ( "ketotic diet" ) ) )

1. ***Search strategy in Web of Science***

"Diabetes Mellitus" (Topic) or ( "Noninsulin-Dependent" AND "Diabetes Mellitus" ) (Topic) or ("Diabetes Mellitus" AND "Ketosis-Resistant") (Topic) or "Ketosis-Resistant Diabetes Mellitus" (Topic) or ("Diabetes Mellitus" AND "Non-Insulin-Dependent") (Topic) or "Non-Insulin-Dependent Diabetes Mellitus" (Topic) or ("Diabetes Mellitus" AND stable) (Topic) or ("Diabetes Mellitus" AND "Type II") (Topic) or NIDDM (Topic) or "Type 2 Diabetes Mellitus" (Topic) or "Noninsulin-Dependent Diabetes Mellitus" (Topic) or "Type 2 Diabetes" (Topic) or T2DM (Topic) AND ketogenic (Topic) or "Ketogenic Diet" (Topic) or (diet AND ketogenic) (Topic) or "keto diet" (Topic) or "ketogenous diet" (Topic) or "ketotic diet" (Topic)

1. ***Search strategy in Embase***

'diabetes mellitus'/exp OR 'diabetes mellitus' OR 'diabetes mellitus':ti,ab,kw OR ('noninsulin-dependent':ti,ab,kw AND 'diabetes mellitus':ti,ab,kw) OR ('diabetes mellitus':ti,ab,kw AND 'ketosis-resistant':ti,ab,kw) OR 'ketosis-resistant diabetes mellitus':ti,ab,kw OR ('diabetes mellitus':ti,ab,kw AND 'non-insulin-dependent':ti,ab,kw) OR 'non-insulin-dependent diabetes mellitus':ti,ab,kw OR ('diabetes mellitus':ti,ab,kw AND stable:ti,ab,kw) OR ('diabetes mellitus':ti,ab,kw AND 'type ii':ti,ab,kw) OR niddm:ti,ab,kw OR 'type 2 diabetes mellitus':ti,ab,kw OR t2dm:ti,ab,kw OR 'noninsulin-dependent diabetes mellitus':ti,ab,kw OR 'type 2 diabetes':ti,ab,kw OR 'adult-onset diabetes mellitus':ti,ab,kw OR ('diabetes mellitus':ti,ab,kw AND 'insulin-dependent':ti,ab,kw) OR ('diabetes mellitus':ti,ab,kw AND 'insulin dependent':ti,ab,kw) OR 'insulin-dependent diabetes mellitus':ti,ab,kw AND 'ketogenic diet'/exp OR 'ketogenic diet' OR (diet:ti,ab,kw AND ketogenic:ti,ab,kw) OR 'keto diet':ti,ab,kw OR 'ketogenous diet':ti,ab,kw OR 'ketotic diet':ti,ab,kw

Figure 1S: Forest plot detailing weighted mean difference and 95% confidence intervals for the impact of VLCKD on HOMAIR.


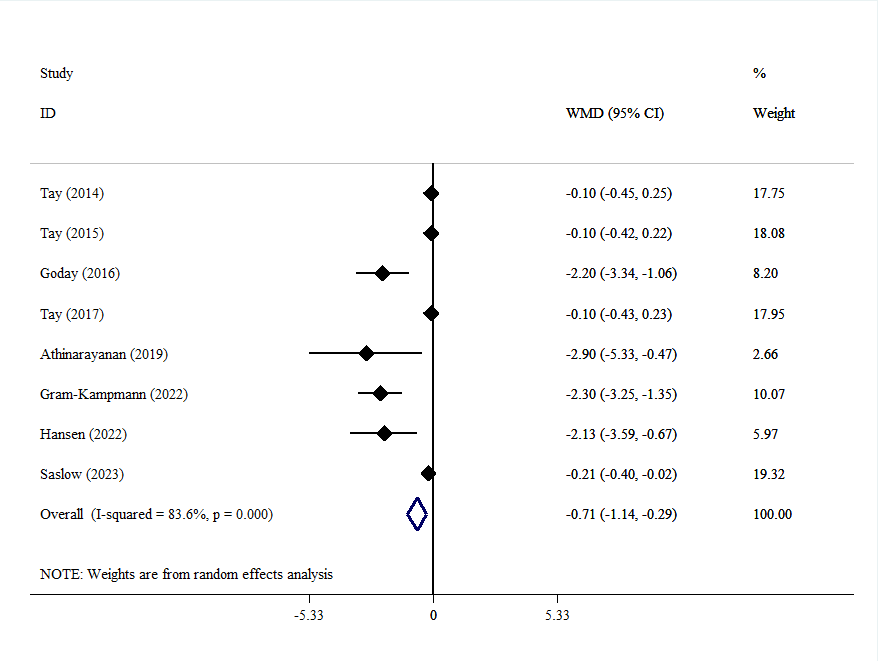


**Figure2s:** **Forest plot detailing weighted mean difference and 95% confidence intervals for the impact of VLCKD on insulin.**

**
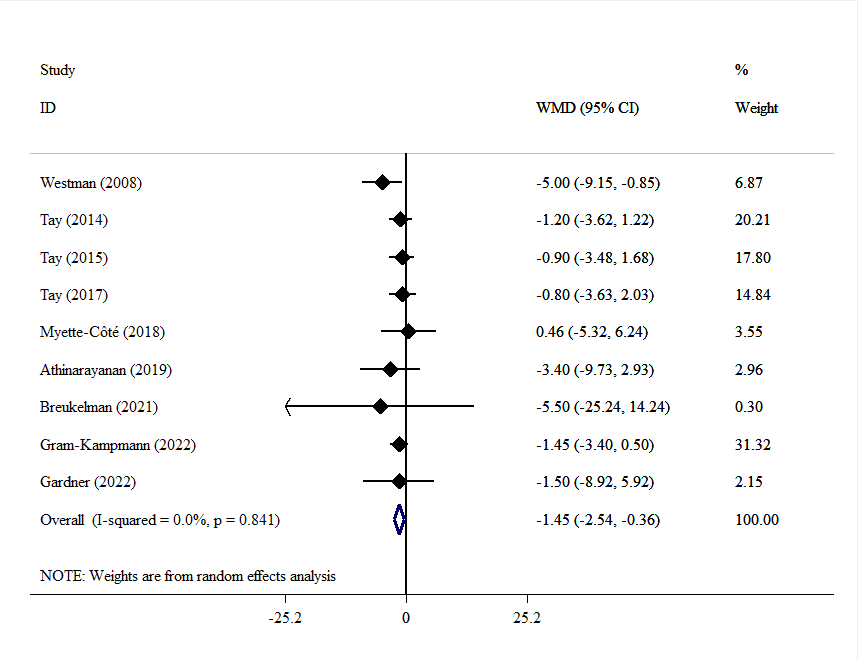
**

**Figure 3S: Funnel plots detailing publication bias in the studies selected for TC**


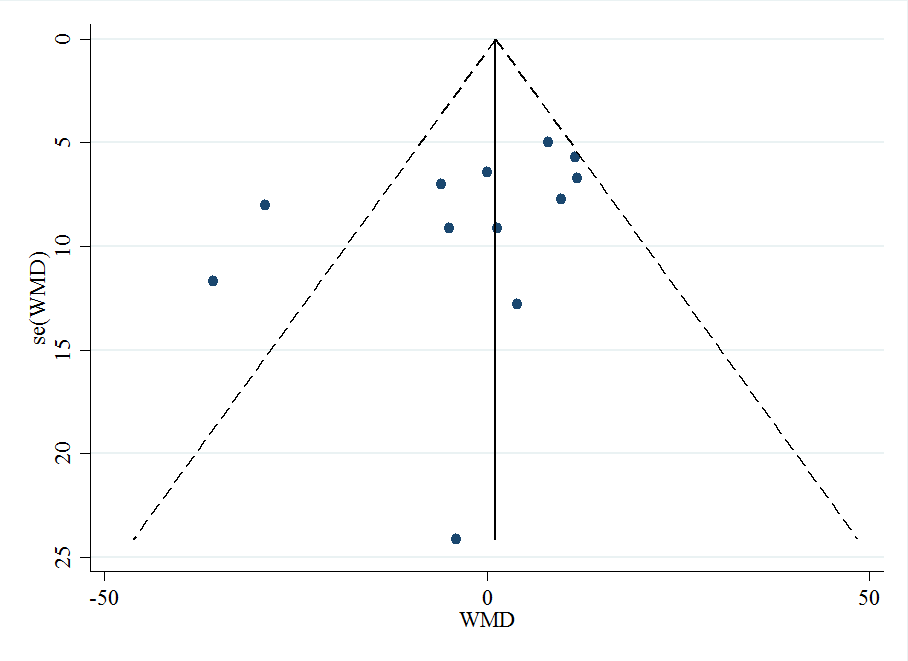


**Figure 4S:** Forest plot detailing weighted mean difference and 95% confidence intervals for the impact of VLCKD on LDL.


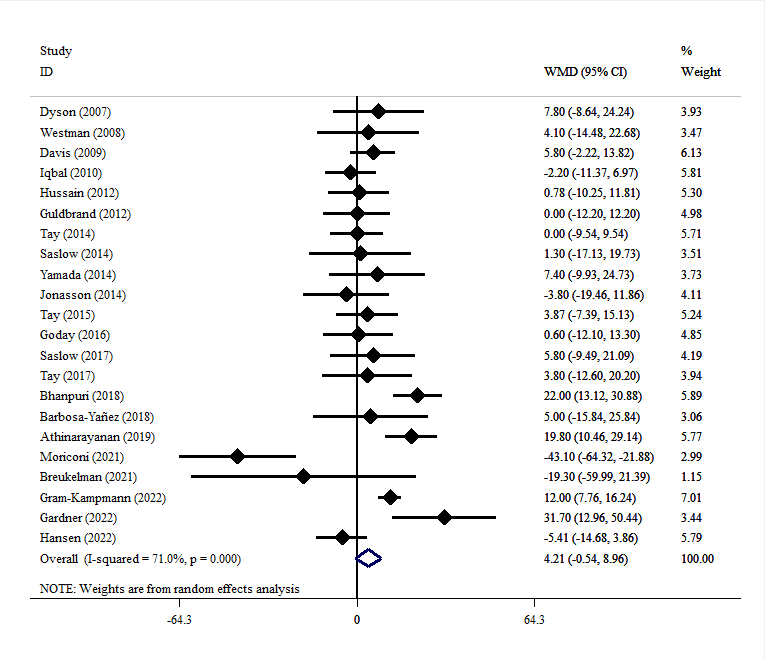


**Figure 5S: Forest plot detailing weighted mean difference and 95% confidence intervals for the impact of VLCKD on HDL.**

**
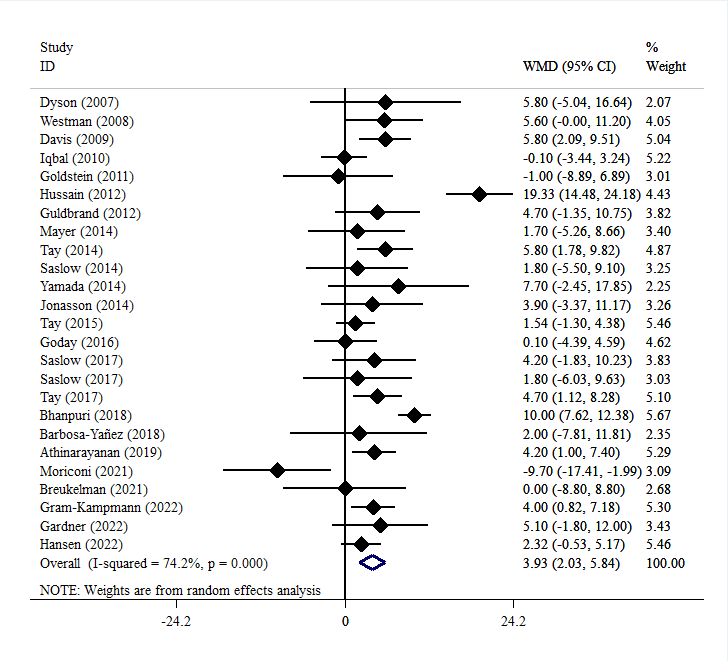
**

**Figure 6S: Forest plot detailing weighted mean difference and 95% confidence intervals for the impact of VLCKD on DBP.**

**
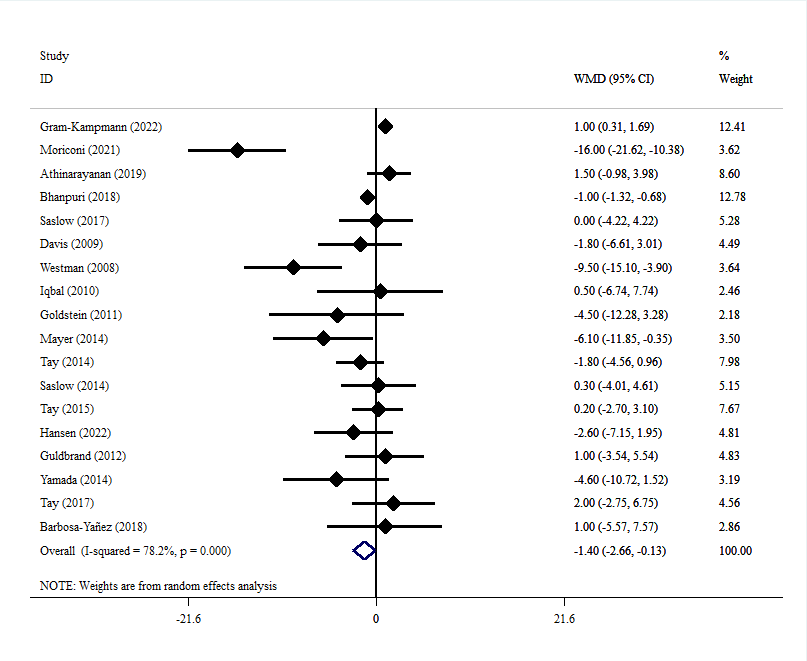
**
